# Editorial

**Published:** 1864-11

**Authors:** 


### Editorial.

GALACTOGOGUES.—There are few physicians in active practise who do not meet with mothers, in whose breasts the secretion of milk becomes too scanty for the wants of the child, and occasionally one, in whom there is a uniform tendency manifested by the mammary glands to stop the secretion altogether at an early period of lactation. To remedy this, many remedies have been tried, but generally with little or no benefit. Among the agents most frequently resorted to, are the fermented drinks, as beer, ale, porter, &c. We have inquired, closely, concerning the effects of these agents on a considerable number of nursing women, but have never yet seen a case in which they caused a perceptable increase in the quantity of milk secreted. As all experiments with alcoholic liquors, appear to show conclusively that while present in the blood, the sum total of secretions and eliminations is diminished; we could not expect them to act the part of galactogogues. Recently, Dr. SKINNER, of Liverpool, has claimed decided success in promoting the secretion of milk by faradization, or the passage of gentle electric currents through the mammary glands, once a day, or once in two or three days, according to the degree of deficiency in secretion. The remedy is simple, easy of application, and certainly worthy of a fair trial. If any internal remedies were to be used, we would suggest the compound syrup of the hypophosphites, especially when the mother is more or less anemic.

THE ILLINOIS STATE MEDICAL SOCIETY.—COMITTEE ON DISEASES OF THE EYE.—The Committee would most respectfully request members of the Society, and of the profession generally, to contribute reports of important cases and papers upon any subject in Ophthalmology

The Chairman of the Committee would especially solicit the record of all facts, relating to epidemics of conjunctivitis in different parts of the State—the condition of the soil and atmosphere—the habits and occupation of the patients, and the severity and number of cases.

We would also solicit the history of cases of injury of one eye followed by sympathetic ophthalmitis in the other eye.

E. L. HOLMES, *Chicago.*
R. E. McVEY, *Waverly.*
J. S. WHITMIRE, *Metamora.*
F. B. HALLER, *Vandalia.*

### CITY MORTALITY.—STATISTICS FOR NOVEMBER.

The assistant health officer, Mr. M. P. Riordan, has just completed his monthly report of the mortality of the City, which is presented below. The whole number of deaths during the month of November was 242, or 28 less than those of the preceding month, and a decrease of 18 from the number of deaths during the corresponding month last year. Small-pox, which was such a fearful scourge in our city in 1863, has not yet prevailed to such an extent as to characterize it as an epidemic. The number of deaths, from this disease during November, was only 11, being a decrease of 7, from those of the same month last year.

DISEASES.

| | | | | | |
|---|---|---|---|---|---|
| Accidents, | 5 | Erysipelas, | 2 | Pleurisy, | 2 |
| Apoplex, | 2 | Gravel, | 1 | Pneumonia, | 2 |
| Consumption, | 42 | Heart Disease, | 4 | Peurperal Fever, | 3 |
| Congestion of Brain, | 4 | Irritation, | 1 | Scalded, | 2 |
| Congestion of Bowels, | 1 | Inflammation of Brain, | 4 | Ship Fever, | 1 |
| Congestion of Lungs, | 2 | Inflammation of Bowels, | 14 | Scrofula, | 3 |
| Congestive Fever, | 1 | Inflammation of Lungs, | 6 | Small-Pox, | 11 |
| Croup, | 15 | Intemperance, | 1 | Stillborn, | 11 |
| Convulsions, | 6 | Jaundice, | 1 | Summer Complaint, | 1 |
| Childbirth, | 2 | Killed, | 4 | Scarlet Fever, | 4 |
| Cramps, | 14 | Lung Fever, | 2 | Typhoid Fever, | 10 |
| Cancer of Stomach, | 1 | Marasmus, | 1 | Typhus Fever, | 3 |
| Diarrhœa, | 6 | Nervous Fever, | 1 | Teething, | 9 |
| Diphtheria, | 7 | Neuralgia, | 6 | Unknown, | 10 |
| Dropsy, | 8 | Old Age, | 1 | Water on the Brain, | 2 |
| Drowned, | 1 | Poisoned, | 1 | | |
| | | | | Total, | 242 |

NATIVITIES.

| | | | | | |
|---|---|---|---|---|---|
| Chicago, | 115 | Wales, | 2 | Prussia, | 1 |
| Colored, | 4 | Bavaria, | 1 | Sweden, | 1 |
| Canada, | 7 | Germany, | 21 | Other States, | 30 |
| England, | 6 | Illinois, | 4 | Unknown, | 4 |
| Ireland, | 29 | Norway, | 7 | | |
| Scotland, | 4 | | | Total, | 242 |

AGES.

| | | | | | |
|---|---|---|---|---|---|
| Under 5 years, | 104 | Over 40 and under 50, | 19 | Over 90 and under 100, | 2 |
| Over 5 and under 10, | 19 | Over 50 and under 60, | 14 | Unknown, | 3 |
| Over 10 and under 20, | 12 | Over 60 and under 70, | 12 | Stillborn, | 12 |
| Over 20 and under 30, | 14 | Over 70 and under 80, | 5 | | |
| Over 30 and under 40, | 26 | | | Total, | 242 |

COMPARATIVE TABLE.

The following comparative tables show the number of deaths in each division of the City, for the months of November in this and the preceding year:—

| *November*, 1864. | | *November*, 1863. | |
|---|---|---|---|
| North Division, | 79 | North Division, | 61 |
| South Divison, | 89 | South Division, | 125 |
| West Division, | 73 | West Division, | 74 |
| Unknown, | 1 | | |
| | | Total, | 260 |
| Total, | 242 | | |

---

Messrs. Editors.—With the view of making the list of Permanent Members of the American Medical Association a correct one, the undersigned desires information from the members relative to any changes or deaths which may have occurred since the publication of the volume for 1863. Response should be made immediately, as the new volume is nearly ready for the binder.

Wm. B. Atkinson, M.D.,
*Philadelphia, Dec.* 8, 1864. *Permanent Secretary.*

---

Catheterism of the Duodenum and Jejunum.—Mr. Blanchet, in a paper presented to the *Academie des Sciences*, mentions four cases in which this operation was successfully effected, for the purpose of expelling foreign bodies engaged in the digestive tube, or of overcoming intestinal occlusion. The feelings of the patient seemed to afford sufficient proof that the sound penetrated beyond the pylorus, and experiments on the dead subject prove that the instrument can be introduced without serious difficulty through the duodenum into the first part of the jejunum. The author suggests that this will prove a useful method for distinguishing strictures, tumors, occlusion, and foreign bodies of the intestinal canal, and for introducing remedies or food beyond the pyloric orifice of the stomach, when that organ, from a state of disease, cannot tolerate them. The flatus, which sometimes accumulates in the intestine, giving rise to dangerous symptoms, may likewise be evacuated by the same means.—*Australasian Med. and Surg. Review.*

---

Obstinate Constipation.—M. Homolle has found the following powder efficacious in two cases, where obstinate constipation had raised the question of operation for artificial anus:—Powdered strychnine, one fiftieth of a grain; powdered nux vomica, one-fifth of a grain; calcined magnesia, six grains; mix. One powder a day at first then two, and finally three per diem. In both cases the bowels were moved, and the symptoms of suspected internal strangulation disappeared.

---

Professor Trousseau on Aphasia.—This popular professor, in one of his late clinical lectures at the Hotel Dieu, of Paris, dwelt on a peculiar complaint, the symptoms of which are the inability in the patient pronouncing certain words, and of expressing his thoughts, retaining at the same time the full use of his intellectual faculties. M. Broca, who first directed attention to this malady, called it *aphœmia*, which term M. Trousseau, aided by the celebrated philologist, Littre, considers wrong, as it really means "bad repute." *Aphasia* is better.

